# Assessment of Patient Risk Profiles by a Male Sexual Health Direct-to-Consumer Prescription Platform: A Cross-Sectional Study

**DOI:** 10.1089/tmr.2023.0010

**Published:** 2023-06-13

**Authors:** Moritz von Büren, Christian Wülfing, Daniel Schlager, Max Michael Träger, Marcel Daoud, Florian Schröder, Sabine D. Brookman-May, Christian Gratzke, Johannes von Büren

**Affiliations:** ^1^ Department of Urology, University of Freiburg, Freiburg, Germany.; ^2^ Department of Urology, Asklepios Klinik Altona, Hamburg, Germany.; ^3^ Wellster Healthtech Group, Munich, Germany.; ^4^ Technical University of Munich, Munich, Germany.; ^5^ Janssen Research and Development, LLC, Spring House, Pennsylvania, USA.; ^6^ Department of Urology, Ludwig Maximilian University of Munich, Munich, Germany.

**Keywords:** telemedicine, safety, andrology

## Abstract

**Background:**

Direct-to-consumer (DTC) online prescription platforms (OPP) for sexual health represent a potential paradigm shift in the diagnosis and treatment for sexual dysfunctions in the way men seek care. Knowledge of patients' risk profile using these platforms is limited.

**Aim:**

To assess risk profiles of patients reaching out to health care professionals through their DTC.

**Methods:**

Anonymized data originally collected between February 2021 to May 2022 by a DTC platform in the men's health care space were retrospectively analyzed. Data included the content of patient requests through a communication function, as well as the corresponding responses by the attending physician on staff. Each request was then assessed by two independent urologists and categorized by the level of the risk profile as well as the need to refer the patient to further medical evaluation.

**Results:**

Of 585 patient requests, 531 (90.8%) were classified as low risk. In the high-risk group, 32 patients were recommended to schedule an urgent appointment at a specialist. Only three patients (0.5%) were advised to seek emergency care. The overall referral rate for both risk groups was 52.3%. The requests of 279 patients (47.7%) were assessed as digitally treatable. Almost all patients who were digitally treatable were low risk. Side effects accounted for only 9.6% of all requests in the low-risk group, compared with 46.3% in the high-risk group.

**Conclusion:**

Overall, low-risk levels in the requests of patients using a DTC platform were reported, with almost half of them suitable to be solved digitally, whereas the other half required referral to an in-person specialist.

## Introduction

Telemedicine is a collective term for different medical care concepts that embrace the principal concept of providing medical attention in the form of diagnostics and therapy through a geographical distance or time offset.^1^ Therefore, different means of communication, such as video or web-based systems, can be used.^2^ There are constant discussions about risk and benefit issues concerning telemedical concepts.^3,4^ Parallel to the efforts of the public health sector to implement a secure and stable telematics infrastructure, there are several private companies providing online health services.^5^

These companies and their medical personnel treat millions of patients exclusively digital.^6^ Although telemedical concepts and direct-to-consumer (DTC) companies in a few medical fields have been reviewed for their risk potential and effectiveness,^7,8^ there are limited data on the use of telemedicine in the field of sexual medicine. Thus, no guidelines for telemedical diagnosis, treatment, and prevention of sexual health indications have been established yet.^9,10^

We, therefore, examined an anonymized data set provided by Wellster Healthtech Group, the provider of “www.gospring.de,” a German DTC prescription platform for men's sexual health. The website offered in 2021–2022 an “ask a doctor” function on a written basis for patients concerning questions in the urological sector. This function was free of charge, and the patients' questions were answered by an attending urologist of the Wellster Healthtech Group within 2 days.

The aim of this study was to evaluate the content and severity of patient requests sent through a direct patient–physician channel and the response of the attending physicians, providing insight into potential health risks for patients reaching out to health care professionals for health-related questions through their DTC platform.

## Methods

### Study design and data collection

This cross-sectional study was conducted with anonymized data supplied by Wellster Healthtech Group, the provider of “www.gospring.de,” a DTC platform for men's sexual health. The platform is registered in Germany and advertised on internet search engines, digital media, and commercial spots for self-payment treatment indications. Patient data were collected through structured questionnaires. After evaluation of the diagnosis and potential contraindications, patients received prescriptions for medication or alternative treatment options. In addition to the prescribed treatment, patients could reach out to physicians through the “ask a doctor” function, through e-mail, on the website and in their user account. This allowed patients to write questions regarding their health. These questions were then answered directly by the attending urologist. This service was free of charge for patients.

Anonymized patient data for analysis was provided by the DTC as described.^11^ All requests from patients and the answers from the attending urologist were extracted from the medical data system. Requests were sent between February 2021 and May 2022. All research was carried out in accordance with the Code of Ethics of the World Medical Association (Declaration of Helsinki) and its later amendments. Informed consent was received from all patients. Before initiation of the study, the local ethics authority (Ethikkommission der University of Freiburg) revised the project design and waived the need for approval (Ref. No. 21-1688-retro).

### Request and response assessment

In the first step of assessment, the potential harm for patients and urgency for admission were analyzed. Using predefined categories, two urologists independently assessed patient requests, the answers provided by the attending physician, and the required action. When the independent urologists differed in their assessment, risk classification was then conducted by an experienced senior urologist, and consensus was found.

Patient requests were classified into the following categories: (1) no potential harm; (2) low potential harm, no life-threatening risk within a week; (3) high potential harm, but no life-threatening risk within a week; and (4) life-threatening risk within a week. These categories were grouped in a low-risk (category 1, P^1^) and high-risk (categories 2–4, P^2^). In addition, the required action by a treating physician was assessed by two independent urologists as follows: (1) no action required, telemedical consultation sufficient to solve the request; (2) elective appointment at offline specialist; (3) urgent consultation of a specialist within a week; and (4) emergency admission.

In the second step of assessment, the requests and respective recommendations made by the “real world” attending urologist on the platform were categorized. Requests were grouped into contraindication, drug interaction, side effect, diagnostic, treatment success, and other. The attending physician's referrals to an outpatient physician were counted and then sorted by their medical specialty, including urology, cardiology, psychology, family practice, and other. Finally, the attending physician's recommendations were categorized by the main focus of the doctor's response: medical advice, treatment start, treatment adjustment, treatment stop, treatment continuation, outpatient doctor referral, and other.

### Statistical analysis

Data management and analysis were performed using GraphPad Prism software version 8 (GraphPad Software, San Diego, CA, USA). In Tables 1–3, the number in parentheses indicates the percentage of a category within the parameters: indication, subject of request, assigned outpatient doctor and main focus of doctor's response, independently in groups P^1^ and P^2^. A chi-square test was used for the analysis of comparisons of parameters between P^1^ and P^2^. All statistical tests were two-sided, and the *α*-level was set at 5% (*p* ≤ 0.05).

**Table 1.** Characterization of Patient Requests to Telehealth Providers According to Risk of Harm

Patients	Subgroup by risk assessment		All	
	*P*^1^; *n* = 531	*P*^2^; *n* = 54	*P*^1^ + *P*^2^; *n* = 585	
Group	Low risk	High risk	Both	
Parameter	*n* (%)	*n* (%)	*n* (%)	*p*
Indication				
ED	391 (73.6)	39 (72.2)	430 (73.5)	0.3673
PE	53 (10.0)	3 (5.5)	56 (9.6)	
Other	87 (16.4)	12 (22.2)	99 (16.9)	
Subject of request				
Contraindication	27 (5.1)	2 (3.7)	29 (5.0)	<0.0001
Drug interaction	92 (17.3)	9 (16.6)	101 (17.3)	
Side effects	51 (9.6)	25 (46.3)	76 (13.0)	
Diagnostics	58 (10.9)	6 (11.1)	64 (10.9)	
Treatment success	226 (42.6)	7 (13.0)	233 (39.8)	
Other	77 (14.5)	5 (9.3)	82 (14.0)	

ED, erectile dysfunction; PE, premature ejaculation.

**Table 2.** Referrals Made to an Outpatient Doctor Through the Attending Physician According to Risk of Harm

Patients	Subgroup by risk of harm		All	*p*
	*P*^1^; *n* = 531	*P*^2^; *n* = 54	*P*^1^ + *P*^2^; *n* = 585	
Group	Low risk	High risk	Both	
Parameter	*n* (%)	*n* (%)	*n* (%)	
Assigned outpatient doctor^a^				
None	168 (31.6)	3 (5.6)	171 (29.2)	<0.0001
Urologist	288 (54.2)	32 (59.3)	320 (54.7)	
Cardiologist	72 (13.6)	10 (18.5)	82 (14.0)	
Psychologist	45 (8.5)	3 (5.6)	20 (3.4)	
Family doctor	8 (1.5)	2 (3.7)	10 (1.7)	
Other	10 (1.9)	6 (11.1)	16 (2.7)	

^a^ Required physician could be more than one specialty.

**Table 3.** Main Focus of Attending Physician's Response According to Risk of Harm

Patients	Subgroup by risk of harm		All	*p*
Group	*P*^1^; *n* = 531	*P*^2^; *n* = 54	*P*^1^ + *P*^2^; *n* = 585	
	Low risk	High risk	Both	
Parameter	*n* (%)	*n* (%)	*n* (%)	
Main focus of doctor's response^a^				
Medical advice	99 (18.6)	10 (18.5)	109 (18.6)	<0.0001
Treatment start	64 (12.1)	0 (0.0)	64 10.9)	
Treatment adjustment	115 (21.7)	15 (27.8)	130 (22.2)	
Treatment stop	10 (1.9)	12 (22.2)	22 (3.8)	
Treatment continuation	43 (8.1)	2 (3.7)	45 (7.7)	
Outpatient doctor	183 (34.5)	13 (24.1)	196 (33.5)	
Other	17 (3.2)	2 (3.7)	19 (3.2)	

^a^ Aspects other than the main focus of the response may have been addressed by the doctor and are not included in this analysis.

## Results

### Patient characterization

The majority of patients were diagnosed and treated for erectile dysfunction (ED) or premature ejaculation (PE), whereas less frequent consultations were, for example, andrology-related, ranging from testosterone level to infertility assessment. ED treatments included prescription of phosphodiesterase type-5 (PDE-5) inhibitors (sildenafil, tadalafil, or vardenafil) and lifestyle interventions. PE patients received prescriptions for either local treatment (lidocaine/prilocaine spray) or systemic therapy with dapoxetine.

### Risk assessment

The patient requests (*n* = 585) were analyzed by the two independent urologists according to the need for referral and urgency to present to a specialist. The requests of 279 patients (47.7%) were classified as digitally treatable. An elective appointment with a specialist was required for 271 of the requests (46.3%) and 32 (5.5%) required an urgent appointment with a specialist. Three patients (0.5%) required emergency presentation. Next, patients' risk at the time of the request was assessed as described. Five hundred thirty-one patients (90.8%) were classified as no harm within the next week and, therefore, categorized as low risk. Forty-six patients (7.9%) were classified as suffering potential harm, but no life-threatening event within the next week was likely.

**FIG. 1.**
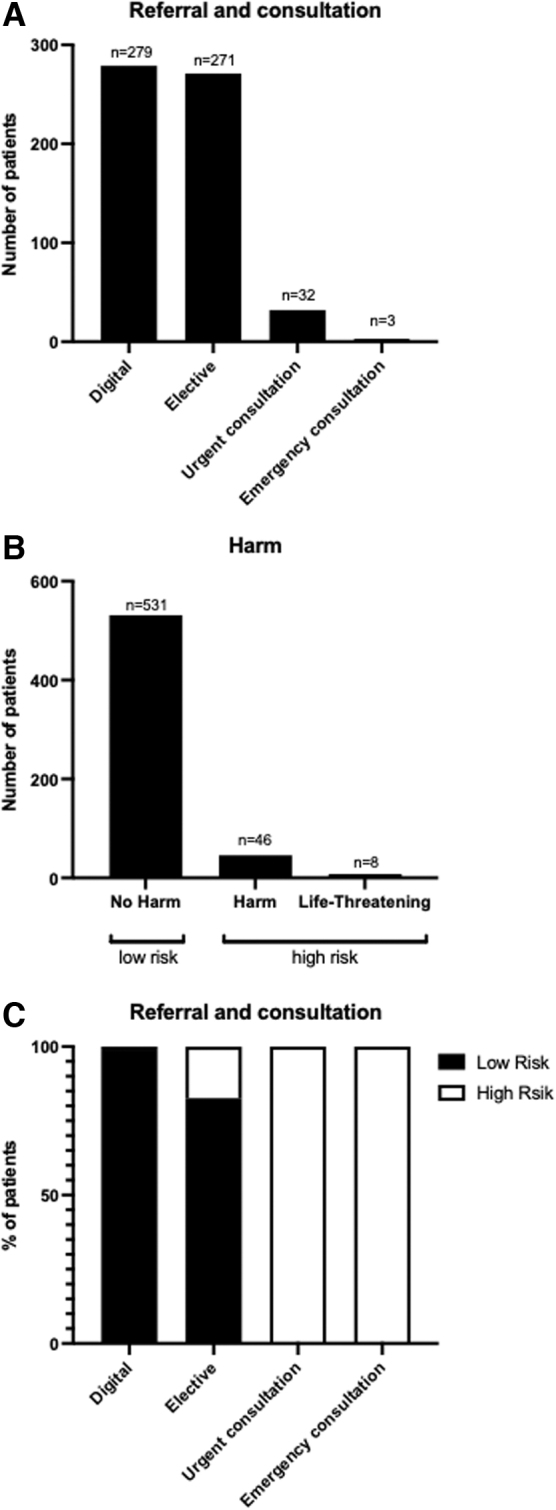
Patients' referral and risk during online request.

Six patients (1.0%) requested help with a potentially life-threatening condition within the next week. Patients with potential harm and potentially life-threatening conditions were categorized as high-risk throughout all further analysis. Almost all patients that were digitally treatable were low-risk patients (*n* = 276), with three patients (1.1%) from the high-risk group. All patients requiring urgent and emergency treatment were from the high-risk group (Fig. 1). In 25 cases, the independent urologist one and two disagreed in their risk assessment, so a senior urologist was consulted for supervision. In 14 (56%) of the 25 cases, the senior urologist agreed with the urologist, who rated the risk of the case under review as “higher” (Supplementary Fig. S1).

### Characterization of patient requests to telehealth providers according to risk of harm

Four hundred thirty (73.5%) patients presented with questions related to ED, 56 (9.6%) related to PE, and 99 (16.9%) with other andrology-related questions, focusing on sexual health or other urological problems (Table 1; for more detail on the ladder group refer to Supplementary Table S1). The most frequent subject of request was treatment success (*n* = 233), followed by drug interaction (*n* = 101), side effects (*n* = 76), and diagnostics (*n* = 64).

After categorizing patients into the aforementioned risk groups, no difference was observed regarding the indication (*p* = 0.3673). However, the subject of request was significantly different between the two risk groups (*p* < 0.0001). The largest relative difference was observed for side effects and accounted for 9.6% of all requests in the low-risk group and 46.3% of the high-risk group. Conversely, requests regarding treatment success were more frequent in the low-risk group (42.6%) than in the high-risk group (13.0%, see Table 1).

### Referrals and responses of the digital attending physician according to risk of harm

Referrals made to an outpatient doctor through the attending physician were significantly different between the two risk groups (*p* < 0.0001). Although almost all of the high-risk requests (94.4%) were referred to an outpatient doctor by the attending physician, referral was only suggested in around two thirds (68.4%) of the low-risk requests (see Table 2). Most recommendations for referrals were for urology (*n* = 320), followed by cardiology (*n* = 82), psychology (*n* = 20), and other specialties (*n* = 16). In around one third of the requests (*n* = 168), no ambulatory referral was provided. Related to the total population (P^1^+P^2^) *n* = 171 patients were treated digitally by the attending physician (Table 2).

The three patient requests that were classified in the high-risk group by the independent urologists were those patients who were not referred to the outpatient doctor by the attending physician (Supplementary Table S2).

Main focus of attending physician's response was significantly different between the two risk groups (*p* < 0.0001). Overall, the primary subject of the doctor's responses were related to referrals to an outpatient doctor (33.5%), followed by treatment adjustment (22.2%) and medical advice (18.6%, see Table 3 and Supplementary Table S3).

## Discussion

Escorted by COVID-19 pandemic, the expansion of the telehealth sector, often lacking governmental control, was immense.^10^ DTC platforms provide health information and additionally promise discrete evaluation and treatment of sexual dysfunctions (such as ED and PE) without requiring an in-person visit. This represents a paradigm shift in care of men with sexual health issues. Overall, low-risk levels are observed in the medical requests of patients in this study. Although almost half of all requests could be addressed digitally, many patients received recommendations for a referral to a resident specialist. Patients' risk issues on digital treatment platforms and their behavior on how to cope with them is completely understudied and referral to resident physicians is unclear. We, therefore, provide the very first insights into patient safety issues and patient request data on a DTC for urological indications.

### Current knowledge of DTC healthtech platforms in urology

At the time of developing this study and article, there were no data available revealing information on DTC platforms in urology regarding patient safety, safety behavior, and quality of care. Previous studies revealed baseline characteristics of patients using online prescription platforms for online ED treatment. We realized that beneficiaries of digital health services were often more treatment naive,^7^ younger (study population had a median age of 49 years), and showed a milder disease presentation, with 61–62% of patients having sustained morning erections compared with the populations described in related ED studies.^11^ No studies thus far have investigated the direct patient inquiries to the care providers of a DTC platform.

In this study, we were able to identify that most of the inquiries dealt with the topic of treatment success (39.8%), followed by drug interactions (17.3%). This indicates that despite patients being able to choose type and treatment dose themselves, there is often still a need for clarification afterward. In this study, the advantage of an online care model can be titration and stepwise selection of the fitting products. This has been proposed for other indications with a longer history of utilizing digital technologies in patient management, such as diabetes mellitus.^12^

Only one study has focused on the feasibility of digital treatment for patients who are treated at large referral centres.^4^ However, those studies are highly limited, as they focus on other patient cohorts aside from patients on DTC platform.^13–15^

### Safety behavior of patients on DTC platforms for urological indications

ED and PE are two urological indications treated mainly by medication, so several safety aspects have to be considered. For PDE-5 inhibitors, a low-risk profile is outlined in the Princeton III Consensus guidelines for cardiovascular conditions.^16^ Those two facts together lead to a potentially considerable risk and adverse events profile for ED prescriptions. Nevertheless, ED is often an early symptom of an underlying chronic condition that requires treatment.^17^ In a conventional system, a certain percentage of patients has to be sent to other specialists, such as cardiologists or psychologists, before or after initiation of a PDE-5 inhibitor therapy.^18^

Up until this study, it was unclear if this recommendation would occur on online platforms, despite the large number of prescriptions in both the United States and Europe.^19^ However, our study revealed a recommendation for seeing a resident specialist in almost all high-risk cases and in 68.4% of all low-risk cases. This is encouraging since in individual cases even rare pathologies can be overlooked by DTC platforms.^4^ Thus, our data show for the first time that referrals to outpatient physicians do occur from the DTC platform side. Whether the patients then make an appointment with an outpatient doctor remains an open question.

When treated online, the patients might place overconfidence in the treatment algorithms and neglect safety issues. We, therefore, are the first to evaluate patient's risk behavior on a DTC healthtech platform for urological indications and how they seek medical help.

### Optimization of direct patient–physician channels on male sexual health DTC platforms

Users of most online platforms for male sexual health in the United States and Europe have the option to send requests directly to the medical team of the respective company to get their medical questions answered. Previously, Broffman et al. analyzed “patient reported” as an unprompted communication initiated by the patient to inform either their telemedicine-affiliated provider or their patient relations team that they experienced a side effect from a prescribed PDE-5.^19^ However, at the time of writing, no one has addressed which requests should be prioritized by the medical affairs team. Our data show that patient inquiries about “side effects” of therapy were most frequently assigned to the “high-risk group” (46.3%). Therefore, we suggest that OPP medical affairs teams should consider establishing a “fast track channel” for inquiries that relate to side effects, providing such patients with quicker feedback on their “high-risk request.”

### Need for further studies on patient characteristics, real-world efficacy, and adverse events

Currently, digital treatment of patients lacks guidelines and scientific evidence, despite the frequent use, in both the United States and Europe.^6,10,13^ Patient characteristics are poorly understood. Furthermore, there is only one study revealing real-world preferences and efficacy of patients using a DTC platform.^11^ However, this knowledge is urgently required to understand which patients can be treated digitally and which contraindications are most prevalent in this population. The study revealed that a higher than expected percentage (48%) of requests could be solved digitally, demonstrating that there was a considerable subgroup of patients who solely received treatment online. Consequently, the question can be raised if and how such a subgroup of patients suitable for digital treatment can be defined—information that could help resident urologists to balance risks when treating patients remotely through telemedicine.

### Need to connect digital and conventional system

While examining the main focus of doctor responses (Table 3), one may be surprised to see that “outpatient doctor” was found to be the main focus more often in the low-risk group (35%) than in the group of high-risk requests (24%). A closer look, however, reveals that almost all of the high-risk requests (94%, Table 1) were referred to a resident specialist in the first place, and the focus of the response subsequently shifted to “treatment adjustment” and “treatment stop” as important follow-ups.

The study also found substantial differences in the doctors' risk assessments, with 68% of the patients referred to an outpatient doctor by the attending physician, compared with only 52% by the two independent urologists. One reason for the higher referral rate could be that online doctors are more likely to use referral as a preventative measure due to their awareness that many patients who seek online treatment may have a higher barrier for consulting an outpatient doctor.^7^ Furthermore, the lack of personal contact between doctor and patient might induce a more tentative referral to avoid forensic difficulties.

It is important to further understand the precise mechanisms how online and offline medicine have to be connected. Certain health problems cannot be solved digitally or require further workup by specialists in-person. To provide continuous patient care, adaptations on both sides will be necessary, with the most important being a common infrastructure.^20^ In Germany, the introduction of a nationwide electronic patient file might pave the way for those kinds of collaborations,^21^ as data can then be exchanged without information loss between different health care players.

### Limitations

Limitations of this study include the cross-sectional and retrospective design. Data were exclusively retrieved from a single telehealth platform and employees of the DTC platform and members of their Medical Board were involved in the export of the data and the preparation of the article. Therefore, other DTC telehealth platforms in urology, including international ones, should be scientifically investigated. The data are exclusively from patients in Germany and no personal health data of patients were collected, the patient cohort from this German DTC although was previously characterized.^7,11^

In addition, there is a risk of selection bias, especially in high-risk patients, as they are more likely to seek help directly in the offline care setting. However, this study aims to address the risk behavior and management of the DTC platform. Furthermore, in the absence of telemedical guidelines, patients are assessed based on the experience of the urologists. In addition, further prospective studies on the short- and long-term patient safety of male sexual health telemedical platforms are needed.

## Conclusion

This cross-sectional study provides the first insights into patient request data on a DTC platform for urological indications. This study reported overall low risk levels in the requests of patients using these platforms for urological conditions. Half of all requests could be solved digitally, which demonstrates the effectiveness of a direct patient–physician channel already used on telemedical platforms today. Nevertheless, risks could also be identified, for example, that side effects occurred particularly frequently as a request topic in the high-risk group. This is precisely why platforms must be particularly careful when dealing with side effects and should implement measures that minimize the risks for these patients.

Interestingly, many patients received recommendations for a referral to a resident specialist. In the high-risk group, there was almost always a referral to an outpatient physician, with a referral rate in nearly two thirds of cases in the low-risk group. These real-world data demonstrate that referrals from patients of the DTC platform to the outpatient setting are indeed made. Therefore, we recommend in the future to examine whether referrals from such platforms to primary care physicians and specialists are actually acted upon by patients. In summary, this study gives first insights about the risk behavior of patients on digital treatment platforms and how these platforms have dealt with it. Nevertheless, further analysis of additional platforms is needed in the future to better understand risks and, thereby, proactively minimize them.

## Authorship Contribution Statement

Conceptualization, methodology, and writing—review and editing by M.v.B., C.W., D.S., M.M.T., M.D., F.S., S.D.B.-M., C.G., and J.v.B. Formal analysis, investigation, and resources M.v.B., F.S., and J.v.B. Writing—original draft by M.v.B., C.W., and J.v.B. Supervision by S.D.B.-M.

## Supplementary Material

Supplemental data

Supplemental data

Supplemental data

Supplemental data

## Abbreviations Used

DTC: direct-to-consumer
ED: erectile dysfunction
OPP: online prescription platforms
PDE-5: phosphodiesterase type-5
PE: premature ejaculation
