# Supplementary material for: Assessment of Patient Risk Profiles by a Male Sexual Health Direct-to-Consumer Prescription Platform: A Cross-Sectional Study
Source: Telemed Rep. 2023 Jun 13;4(1):118–25. doi: 10.1089/tmr.2023.0010 (PMC10282969; doi:10.1089/tmr.2023.0010)
Supplement: Supplemental data [file Suppl_FigS1.docx]

**Supplementary**

**Supplementary Figure 1:** Classification of disputed cases by the experienced senior physician

*****Classification of disputed cases by the experienced senior physician, whether the independent urologist who assessed the case as lower risk, or as higher risk, is upheld
