# Supplementary material for: Assessment of Patient Risk Profiles by a Male Sexual Health Direct-to-Consumer Prescription Platform: A Cross-Sectional Study
Source: Telemed Rep. 2023 Jun 13;4(1):118–25. doi: 10.1089/tmr.2023.0010 (PMC10282969; doi:10.1089/tmr.2023.0010)
Supplement: Supplemental data [file Suppl_TableS1.docx]

|  |  |  |  |  |  |
| --- | --- | --- | --- | --- | --- |

**Supplementary Table 1:** Breakdown characterization of patient requests that are under the parameter indication "other” in Table 1

| Patients |  | **Category “other”** |
| --- | --- | --- |
|  |  | P^1^ + P^2^; n = 99 |
| Groups |  | Both |
| Parameter |  | n (%) |
| Indications / symptoms |  |  |
| Testosterone deficiency |  | 18 (18.2) |
| Penis related diseases* |  | 15 (10.9) |
| Sperma related symptoms** |  | 10 (10.1) |
| Induratio penis plastica |  | 8 (8.0) |
| Androgenetic alopecia |  | 8 (8.0) |
| Anorgasmia |  | 6 (6.1) |
| General health |  | 5 (5.1) |
| Infertility |  | 4 (4.0) |
| Infection |  | 3 (3.0) |
| Other |  | 22 (22.2) |
|  |  |  |

*Phimosis, penis size, penile numbness **Sperm consistency, color, quantity
