# Supplementary material for: Assessment of Patient Risk Profiles by a Male Sexual Health Direct-to-Consumer Prescription Platform: A Cross-Sectional Study
Source: Telemed Rep. 2023 Jun 13;4(1):118–25. doi: 10.1089/tmr.2023.0010 (PMC10282969; doi:10.1089/tmr.2023.0010)
Supplement: Supplemental data [file Suppl_TableS2.docx]

|  |  |  |  |  |  |
| --- | --- | --- | --- | --- | --- |

**Supplementary Table 2:** Classification of three patient requests to the high risk group by the independent urologists without referral to the outpatient doctor through the attending physician

|  | **Indica-tion** | **Summary content of patient request** | |  | **Comment authors regarding decision** | | |  |
| --- | --- | --- | --- | --- | --- | --- | --- | --- |
| Classification of requests* |  |  |  | |  |  |  | |
| High risk group, not referred |  |  |  | |  |  |  | |
| 1 | ED | Overdosing sildenafil 300mg, despite no treatment success | | |  | High risk patient that has to be educated regarding PDE5i dosing | |  |
| 2 | ED | Severe side effects with sildenafil 100mg, afterwards switch to tadalafil with even worse side effects | | |  | High risk patient recommended to reduce dosing of treatment | |  |
| 3 | PE | Oral sex with woman despite application of topical 10% lidocaine spray | | |  | High risk encounter with women, online education to avoid oral sex after application of lidocain spray | |  |

*****Classification of requests as high risk by the two independent urologists and decision not to refer through the attending physician
