# Supplementary material for: Assessment of Patient Risk Profiles by a Male Sexual Health Direct-to-Consumer Prescription Platform: A Cross-Sectional Study
Source: Telemed Rep. 2023 Jun 13;4(1):118–25. doi: 10.1089/tmr.2023.0010 (PMC10282969; doi:10.1089/tmr.2023.0010)
Supplement: Supplemental data [file Suppl_TableS3.docx]

**Supplementary Table 3:** Representative sample of patient requests to the DTC platform

| **Indication** | **Summary content of patient request** |  | **digitally solved or refered** | |
| --- | --- | --- | --- | --- |
| Interaction | May I combine Sildenafil and Tadalafil? | |  | digitally solved |
| Interaction | Are there interactions between Tadalafil and ß-Blockers? | |  | referred |
| Side effects | Unfortunately I get pain in both legs after taking 100 mg sildenafil for several hours  ^.^ | |  | referred |
| pharmaco-kinetics | I take Tadalafil 5mg, when can I expect the maximum effect? | |  | digitally solved |
| pharmaco-kinetics | Which of the PDE5I (Tadalafil vs Sildenafil) is more efficient? Or is the difference just the duration of action? | |  | digitally solved |
| treatment sucess | At the moment I take a pill of 25 mg of Sildenafil with a positiv, but not excellent result. Can I increase the dosing? | |  | digitally solved |
| treatment sucess | I tried both 100 mg of Sildanfil and 20 mg Tadalfil without adequate erection. What shall I do next? | |  | referred |
|  |  | |  |  |
